# Systemic evaluation of inclisiran on the risk of new-onset diabetes and hyperglycemia compared to evolocumab and atorvastatin

**DOI:** 10.3389/fphar.2025.1554631

**Published:** 2025-07-15

**Authors:** Fei Li, Huan Ye, Lingbin Chen, Yuanchun Ma, Sunhui Chen

**Affiliations:** ^1^ Department of Pharmacy, Shengli Clinical Medical College of Fujian Medical University, Fuzhou University Affiliated Provincial Hospital, Fuzhou, China; ^2^College of Pharmacy, Fujian Medical University, Fuzhou, China

**Keywords:** inclisiran, evolocumab, atorvastatin, hyperglycemia, new-onset diabetes

## Abstract

**Background:**

Inclisiran is an siRNA-based cholesterol-lowering drug with N-acetylgalactosamine carbohydrate (GalNAc) and is used for the treatment of hypercholesterolemia or dyslipidemia. It reduces LDL-C by 50%, with a convenient dosing schedule and fewer adverse events. Unlike statins, inclisiran has not been associated with an increased risk of muscle or liver adverse events in clinical studies. This favorable safety profile makes inclisiran a valuable alternative for patients who are intolerant of statins due to muscle or hepatic side effects. However, its impact on glycemic control and diabetes risk is unclear and understudied.

**Methods and results:**

The US Food and Drug Administration Adverse Event Reporting System (FAERS) study analyzed hyperglycemia and diabetes risks for inclisiran, atorvastatin, and evolocumab. Data from 2021 to 2024 were assessed for Medical Dictionary (MedDRA) terms, and SAS 9.4 with a reporting advantage ratio (ROR) and Bayesian credible interval progressive neural network (BCPNN) was used for analysis. Systematic review and meta-analysis were conducted using PubMed, Embase, and specific search terms. Two research workers extracted data independently, and the study quality was assessed with the Cochrane and Newcastle–Ottawa scales. RevMan 5.4 and Stata 18.0 were used for analyses. Ethical approval was waived due to the use of public, anonymous data. From 2015 Q1 to 2024 Q1, 12,821,285 adverse events were reported in FEARS, with 3,375 inclisiran, 126,620 evolocumab, and 42,228 atorvastatin cases. Atorvastatin had a higher ROR for type 2 diabetes (195.03) than inclisiran (0.95) and evolocumab, but it was not statistically significant. Glucose intolerance and blood glucose issues showed weak signals for inclisiran and atorvastatin. A literature search yielded 16 relevant articles, including six cohort studies and 10 RCTs, totaling 297,863 patients. The incidence of new-onset diabetes was higher with atorvastatin than with inclisiran, placebo, and evolocumab. The SUCRA rankings were atorvastatin > inclisiran > placebo > evolocumab for new diabetes incidence.

**Conclusion:**

The FAERS study and meta-analysis indicate that inclisiran may carry a lower risk of new-onset diabetes than atorvastatin, warranting further investigation into inclisiran’s impact on glycemic control.

## Introduction

Inclisiran is a novel cholesterol-lowering small interfering RNA (siRNA) combined with three-stranded N-acetylgalactosamine carbohydrate (GalNAc) and is used as an adjunctive dietary therapy for primary hypercholesterolemia or mixed dyslipidemia (Lamb, 2021). Clinical trials have shown that subcutaneous inclisiran effectively reduces LDL-C levels by approximately 50%, with the advantages of a favorable administration regimen (1–90–180 days) and a lower incidence of adverse events (AEs), which provide better compliance (Sinning and Landmesser, 2020; Merćep et al., 2022).

Studies have shown that 3-hydroxymethylglutaryl-coenzyme A reductase (HMG-CoA) inhibitors (e.g., atorvastatin) adversely affect glycemic control and increase the risk of new-onset diabetes mellitus (NOD) by 9%–46% (Laakso and Fernandes Silva, 2023; Cederberg et al., 2015). Recently, a real-world pharmacovigilance study also demonstrated that PCSK9 inhibitors (e.g., evolocumab) have adverse effects on glycemic control (Goldman et al., 2022). These findings link the use of certain lipid-lowering drugs with the risk of impaired glycemic control and NOD (Kosmas et al., 2018), whereas diabetes is a well-established cardiovascular risk factor that accelerates atherosclerosis and microvascular and macrovascular lesions (Kosmas et al., 2020). The risk of hyperglycemia and new onset of diabetic AEs in patients receiving inclisiran remains unknown and has rarely been studied in pre-approved clinical trials. Unlike statins, inclisiran has not been associated with an increased risk of muscle or liver AEs in clinical studies. This favorable safety profile makes inclisiran a valuable alternative for patients who are intolerant of statins due to muscle or hepatic side effects. However, its impact on glycemic control and diabetes risk is unclear and understudied.

Here, we retrospectively analyzed the data on glycemic control of inclisiran, evolocumab, and atorvastatin in the US Food and Drug Administration Adverse Event Reporting System (FAERS) and combined a meta-analysis of randomized controlled trials (RCTs) to comprehensively evaluate the occurrence of AEs of diabetes to provide a useful reference for clinical practice.

## Materials methods

### Pharmacovigilance study

A pharmacovigilance study was conducted using the FAERS database to assess the risk of target drugs for hyperglycemia and NOD in a large population. All AE reports including inclisiran from 2021 Q1 to 2024 Q1 (first marketed in the EU in December 2020) and atorvastatin and evolocumab from 2015 Q1 to 2024 Q1 (evolocumab was first marketed in Japan in March 2015) were screened. Each report in the FAERS database was coded with preferred terms (PTs) including “type 2 diabetes,” “elevated blood glucose,” “impaired glucose tolerance,” and “elevated glycated hemoglobin” from the Medical Dictionary (MedDRA) version 27.0. We used SAS 9.4 for statistical analysis (SAS software is one of the statistical analysis software applications recommended by FDA FAERS database mining), using the reporting advantage ratio (ROR) and Bayesian credible interval progressive neural network (BCPNN) methods as the target ADE signal mining approaches. These are common techniques for detecting disproportionality in passive monitoring databases. A statistical association between a drug and an AE was considered significant only when both statistical measures, ROR and IC_025_, exceeded their respective significance thresholds (95% confidence interval greater than one and positive IC_025_ value). Adverse drug event associations were not considered statistically significant only when the significance threshold was exceeded (Goldman et al., 2022).

### Systematic evaluation and meta-analysis

#### Literature search and study selection

We searched the electronic literature from when it was built to August 18, 2024; the search terms were “Inclisiran,” “Evolocumab,” “Atorvastatin,” “New onset diabetes,” “Randomized Controlled Trials as Topic,” and “Cohort study” in PubMed, Cochrane Library, Embase, Web of Science, and the US clinical trial registration platform, and literature search was conducted by combining the subject words and free words. According to the inclusion criteria, the study subjects were patients with confirmed hyperlipidemia; the intervention drugs were inclisiran, evolocumab, and atorvastatin; and the control group was the RCT and cohort study taking placebo. Two investigators (Ye and Li) independently extracted and processed information data on the literature of the article, such as the author, title, published year, journal, cases in the trial and control groups, and the number of new diabetes cases in the test and control groups. Studies with case reports, experience summaries, conference abstracts, and incomplete study data or articles were excluded, and literature included in the meta-analysis was determined after reaching consensus through discussion.

#### Evaluation of literature quality

Two investigators independently evaluated the literature quality, and the RCT was evaluated in strict accordance with the Cochrane risk of bias tool. The evaluation indicators included seven items in a total of six aspects: selection bias (random sequence generation and allocation concealment), reporting bias (selective reporting findings), implementation bias (blinded to investigators and subjects), detection bias (blind evaluation of study outcome), loss-to-visit bias (incomplete outcome data), and other bias (other sources of bias). Each entry was evaluated as “low risk,” “high risk,” or “unclear,” and then the risk of bias was drawn using RevMan 5.4 software. The cohort study was evaluated in strict accordance with the Newcastle–Ottawa Scale (NOS) for selection, comparability, and outcome, with a total score of 9; the literature with a score of 6 was generally considered high-quality literature. If there was any disagreement, the third investigator will intervene in the discussion and make the final decision.

#### Statistical treatment

The results of the meta-analysis were statistically analyzed on the data using the RevMan 5.4 software. Outcome measures were dichotomous variables, using the odds ratio (OR) as the efficacy analysis statistic with 95% confidence intervals (95% CIs). Heterogeneity among studies and outcomes was included using the chi-square values test. If I2 > 50% or p 0.1, there was significant heterogeneity among the results of the index, which was analyzed using the random effects model, sensitivity analysis was performed to find the sources of heterogeneity, and subgroup analysis of factors was performed, leading to heterogeneity if necessary; if I2 at 50% and p > 0.1, a fixed-effects model was used to produce a forest plot. Reticular meta-analysis was performed using Stata 18.0 software to plot the mesh evidence, and for studies with closed rings, inconsistency testing and model species were selected. The index of this study was dichotomous variable, so the OR combined statistics were used, and 95% CI was calculated with p < 0.05; the cumulative ranking curve of each intervention (the surface under the cumulative ranking curve, SUCRA) and the ranking plot were drawn.

### Ethical statement

As the FAERS database and RCTs are open to the public and patient records are anonymous and de-identified, ethical permission and informed consent were not required for this study (Zhou et al., 2023).

## Results

### Descriptive results of the pharmacovigilance analysis

From 2015 Q1 to 2024 Q1, a total of 12,821,285 patient AEs were reported in the FEARS database, including 3,375 for inclisiran, 126,620 for evolocumab, and 42,228 for atorvastatin. The median age of patients in the three groups was similar (IQR: 66∼70 years), and the proportion of women (48.39%∼53.95%) was higher than that of men. The number of reported cases of evolocumab and atorvastatin (53.56% and 53.38%) was higher than that of consumers (47.76% and 45.45%), and the number of reported cases (57.04%) was higher than that of health professionals (42.79%). A total of 1,228 (36.39%) AEs reported the time of AEs, 10,749 (8.49%) and 9,752 (23.09%), and the highest proportion between days 0–30 after administration (26.19%, 4.5%, and 11.67%, respectively). The results are shown in Table 1.

**TABLE 1:** Demographic characteristics of patients treated with inclisiran, evolocumab, and atorvastatin in the FAERS database.

Categories	Groups	Inclisiran (n = 3375)	Evolocumab (n = 126620)	Atorvastatin (n = 42228)
Age (years)	18–44 (%)	47 (1.39)	19719 (1.56)	1666 (3.95)
	45–64 (%)	364 (10.79)	30519 (24.10)	12866 (30.47)
	65–74 (%)	732 (21.69)	32738 (25.86)	9654 (22.86)
	≥75 (%)	428 (12.68)	23693 (18.71)	7604 (18.01)
	Missing (%)	1804 (53.45)	37699 (29.77)	10438 (24.72)
Gender	Female (%)	1677 (49.69)	68315 (53.95)	20434 (48.39)
	Male (%)	1234 (36.56)	50284 (39.71)	16626 (39.37)
	Missing (%)	464 (13.75)	8021 (6.33)	5168 (12.24)
Reporter	Health professional (%)	1444 (42.79)	67819 (53.56)	22542 (53.38)
	Consumer/Lawyer (%)	1925 (57.04)	57944 (45.76)	19194 (45.45)
	Missing (%)	6 (0.18)	857 (0.68)	492 (1.17)
Reporting region	Americas (%)	2925 (86.67)	122410 (96.68)	21837 (51.71)
	Europe (%)	283 (8.39)	1800 (1.42)	16169 (38.29)
	Asia (%)	63 (1.87)	1088 (0.86)	2376 (5.63)
	Australia (%)	13 (0.39)	232 (0.18)	505 (1.20)
	Africa (%)	33 (0.98)	17 (0.01)	317 (0.75)
	Missing (%)	58 (1.72)	1073 (0.85)	1024 (2.42)
Time of AEs (days)	0–30d (%)	884 (26.19)	5702 (4.50)	4928 (11.67)
	31–60d (%)	63 (1.87)	812 (0.64)	817 (1.93)
	61–90d (%)	67 (1.99)	564 (0.45)	529 (1.25)
	91–120d (%)	98 (2.90)	374 (0.30)	373 (0.88)
	121–150d (%)	13 (0.39)	279 (0.22)	252 (0.60)
	151–180d (%)	18 (0.53)	275 (0.22)	220 (0.52)
	181–360d (%)	56 (1.66)	1021 (0.81)	608 (1.44)
	>360d (%)	29 (0.86)	1722 (1.36)	2025 (4.80)
	Missing (%)	2147 (63.61)	115871 (91.51)	32476 (76.91)

### Disproportionality analysis of diabetes-related adverse events

As shown in Table 2, the analysis of AE associations revealed distinct profiles between treatment groups. For type 2 diabetes AEs, inclisiran showed no statistically significant association [n = 3/3,375, ROR = 0.95 (0.31–2.96), IC_025_ = −1.49]. This lack of association for inclisiran stands in contrast to atorvastatin, which demonstrated a very strong positive association [n = 6,168/42,228, ROR = 195.03 (188.86–201.41), IC_025_ = 6.78]. For impaired glucose tolerance (IGT) AEs, the association strength observed with inclisiran [n = 5/3,375, ROR = 6.25 (2.60–15.05), IC_025_ = 0.55] was weaker than that observed with atorvastatin [n = 90/42,228, ROR = 9.11 (7.38–11.23), IC_025_ = 2.72]. No statistically significant difference in IGT AE association was found between inclisiran and evolocumab. Analyses identified weak signals for inclisiran treatment-related blood glucose increase [n = 42/3,375, 1.24%, ROR = 2.04 (1.51–2.77), IC_025_ = 0.55] and elevated HbA1c [n = 10/3,375, 0.30%, ROR = 2.72 (1.46–5.06), IC_025_ = 0.36]. Atorvastatin did not show significant signals for these specific glucose-related AEs.

**TABLE 2:** Disproportionality analysis of NOD and hyperglycemic AEs associated with inclisiran, evolocumab, and atorvastatin as compared to the full FAERS database.

ADEs	Inclisiran (n = 3,375)	Evolocumab (n = 126,620)	Atorvastatin (n = 42,228)
Type 2 diabetes mellitus			
No. of cases, n (%)	3 (0.09)	62 (0.05)	6,168 (14.61)
ROR (95% CI)	0.95 (0.31–2.96)	0.51 (0.40–0.65)	195.03 (188.86–201.41)
IC_025_	−0.07 (-1.49)	−0.97 (-1.32)	6.85 (6.78)
Impaired glucose tolerance			
No. of cases, n (%)	5 (0.15)	47 (0.04)	90 (0.21)
ROR (95% CI)	6.25 (2.60–15.05)	2.06 (1.54–2.74)	9.11 (7.38–11.23)
IC_025_	2.64 (0.55)	1.03 (0.58)	3.15 (2.72)
Increased glycosylated hemoglobin			
No. of cases, n (%)	10 (0.30)	311 (0.25)	65 (0.15)
ROR (95% CI)	2.72 (1.46–5.06)	2.58 (2.31–2.89)	1.21 (0.95–1.54)
IC_025_	1.44 (0.36)	1.35 (1.18)	0.27 (−0.09)
Increased blood glucose			
No. of cases, n (%)	42 (1.24)	1,385 (1.09)	358 (0.85)
ROR (95% CI)	2.04 (1.51–2.77)	1.68 (1.60–1.77)	0.98 (0.88–1.09)
IC_025_	1.03 (0.55)	0.74 (0.66)	−0.03 (−0.18)

ROR, A ≥ 3 and 95% CI (lower limit) > 1 will indicate 1 signal; BCPNN, lower limit of credible interval (IC_025_)> 0 indicates 1 signal.

### Results of literature screening by meta-analysis

**FIGURE 1:**
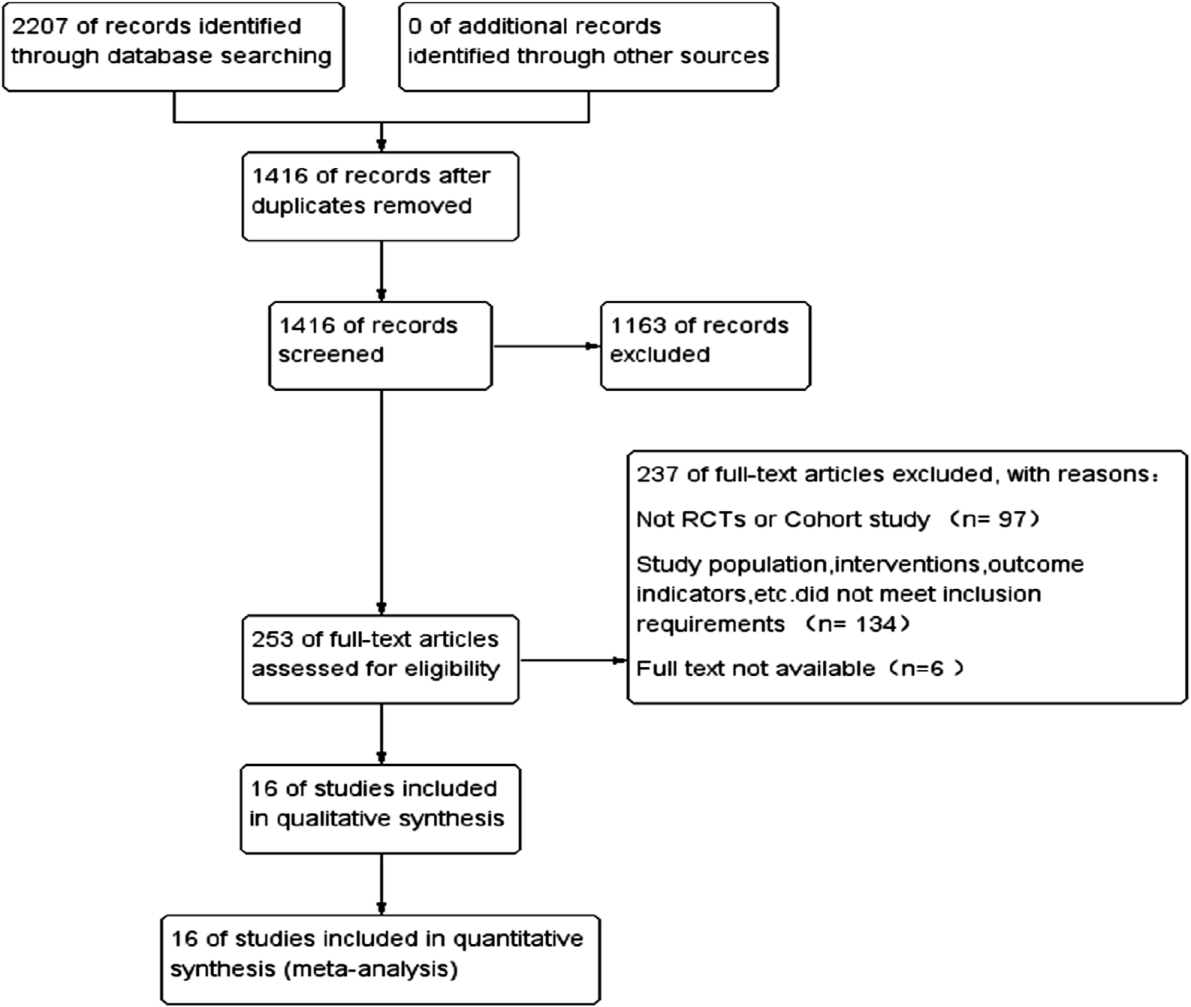
Flow diagram of the network meta-analysis.

Through the search strategy, 2,207 articles were searched in electronic databases, and 1,416 articles were obtained after excluding duplicates. After reading the articles and abstracts, 1,163 articles were selected, including case reports, basic research, systematic review, and meta-analysis; 253 articles were screened for full screening. The full text was read through and screened one by one according to the inclusion and exclusion criteria. Finally, 16 articles met the criteria and were included in this study, involving six cohort studies and 10 randomized controlled trials for a total of 297,863 patients. The literature search and screening process is shown in Figure 1.

### Basic characteristics of the literature in meta-analysis

Basic information of the 16 articles in this study includes the following: study author/study name, year of publication, type of study, intervention measures, number of cases in the experimental group/control group, number of NOD in the experimental group, number of NOD in the control group, ClinicalTrials.gov identifier, including 10 RCT (Mayer et al., 2006; Waters et al., 2011; O’Donoghue et al., 2022; Sattar et al., 2017; Koren et al., 2019), and six retrospective cohort studies (Cederberg et al., 2015; Li et al., 2018; Park et al., 2015; Choe et al., 2014; Culver et al., 2012; Yoon et al., 2016) published from 2003 to 2024, including 297,863 patients, which were studied in both arms. Four studies addressed the comparison of effects on NOD between inclisiran and placebo, four on the effects of evolocumab and placebo, and eight on the effects of diabetes between atorvastatin and placebo. The basic characteristics of the included literature are presented in Table 3.

**TABLE 3:** Basic characteristics of the literature.

Study author/study name	Year of publication	Research design	Intervention study	Number of cases (T/C)/case	Experimental group NOD	Control group NOD	ClinicalTrials.gov identifier
Peter S. Sever	2003	RCT	Atorvastatin	3,910/3,863	154	134	
David D. Waters	2011	RCT	Atorvastatin	1,905/1,898	166	115	
Dukyong Yoon	2016	Cohort study	Atorvastatin	8,342/55,183	35	179	
Ji Young Park	2015	Cohort study	Atorvastatin	409/409	24	13	
H. Cederberg	2015	Cohort study	Atorvastatin	388/6,607	28	386	
E. Y. Choe	2014	Cohort study	Atorvastatin	111/149	13	10	
AL Culver	2012	Cohort study	Atorvastatin	839/143,006	79	9,166	
Li H.	2018	Cohort study	Atorvastatin	9,263/46,442	710	2,216	
Orion10	2020	RCT	Inclisiran	781/778	124	113	NCT03399370
Orion11	2020	RCT	Inclisiran	811/804	92	97	NCT03400800
Orion15	2023	RCT	Inclisiran	255/57	69	15	NCT04666298
SPIRIT	2024	RCT	Inclisiran	273/290	3	2	NCT04807400
Michelle L. O’Donoghue	2022	RCT	Evolocumab	2,155/2,033	90	107	
Naveed Sattar	2017	RCT	Evolocumab	3,298/1,682	127	62	
Michael J. Koren	2019	RCT	Evolocumab	1,255/442	51	19	
MENDEL	2012	RCT	Evolocumab	90/135	1	0	NCT01375777

(Note: RCT, randomized controlled trial; T, test group; C, control group).

### Results of literature quality evaluation in meta-analysis

**FIGURE 2:**
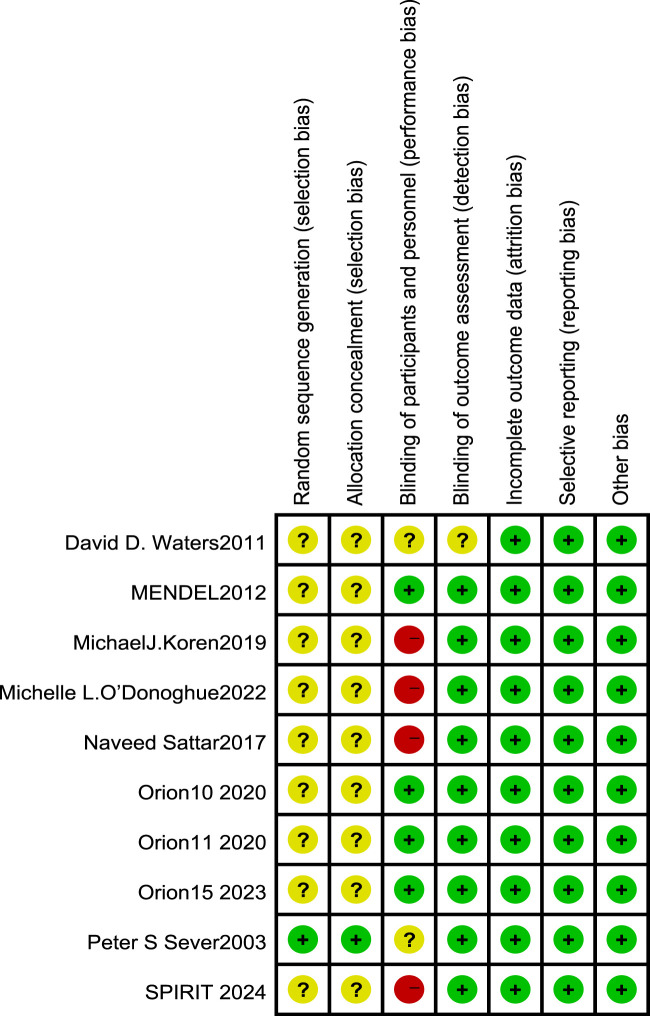
Risk of bias in randomized controlled trials [note: (+), low risk; (−), high risk; (?), NK].

**FIGURE 3:**
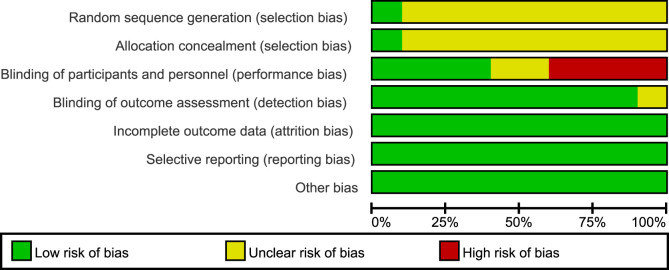
Overall risk of bias assessment diagram of four RCT studies. Note: the bias indicator shows the risk distribution in a percentage manner. The red part indicates the proportion of studies with high risk of bias in all studies, the yellow part indicates the proportion of studies with unclear risk of bias in all studies, and the green part indicates the proportion of studies with low risk of bias in all studies.

All 10 included RCT articles were randomized allocation trials, with only one literature (O’Donoghue et al., 2022) specifically describing the random method, four papers were double-blind, and four papers were not blinded. All RCT study outcomes were complete, the study results were not selectively reported, they had no other sources of bias, and the results of the RCT risk assessment are shown in Figures 2, 3. The NOS score of the six retrospective cohort studies was 19 points, 38 points, and 27 points; three did not truly represent the community population, one did not describe the evaluation method of outcome events, and two had a short follow-up period. The results are shown in Table 4.

**TABLE 4:** Bias risk assessment of cohort studies.

Study, year	Selection				Comparability	Outcome			NOS score
	①	②	③	④	⑤	⑥	⑦	⑧	
Yoon et al. (2016)	1	1	1	1	1	1	0	1	7
Park et al. (2015)	1	1	1	1	2	1	1	1	9
Cederberg et al. (2015)	0	1	1	1	2	1	1	1	8
Choe et al. (2014)	0	1	1	1	2	1	1	1	8
Culver et al. (2012)	0	1	1	1	2	0	1	1	7
Li et al. (2018)	1	1	1	1	2	0	0	1	7

Note: NOS, the Newcastle–Ottawa scale; ① representativeness of the exposed cohort; ② selection of the non-exposed cohort; ③ demonstration that the outcome of interest was not present at the start of the study; ④ demonstration that the outcome of interest was not present at the start of the study; ⑤ comparability of cohorts on the basis of the design or analysis controlled for confounders; ⑥ assessment of outcome; ⑦ was follow-up long enough for outcomes to occur; ⑧ adequacy of follow-up of cohorts.

### Results of the meta-analysis

**FIGURE 4:**
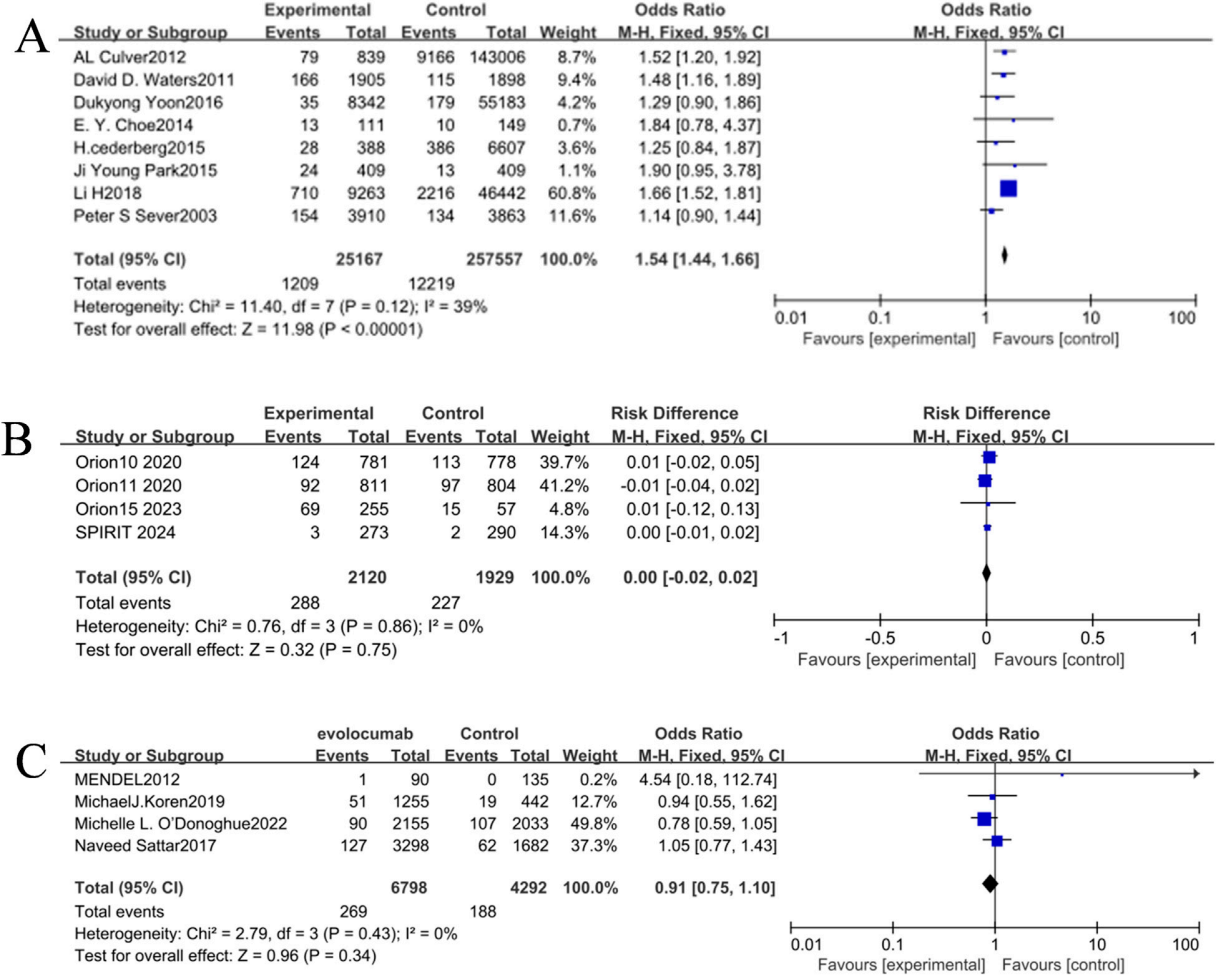
**(A)** Forest plot of the incidence of NOD in the inclisiran and control groups. **(B)** Forest plot of the incidence of NOD in the evolocumab and control groups. **(C)** Forest plot of the incidence of NOD in the atorvastatin and control groups.

Meta-analysis was performed using RevMan 5.4 software. Heterogeneity tests indicated low between-study heterogeneity in the three intervention comparisons (atorvastatin, inclisiran, and evolocumab versus placebo) (p > 0.05, I^2^ < 50%). When p > 0.05 (no significant heterogeneity) and I^2^ < 50% (low heterogeneity), the observed differences among the included studies are primarily attributable to random error rather than variability in true effect sizes. Under this condition, the fixed-effect model, which assumes a common true effect size across all studies, provides a more precise estimate of the pooled effect by accounting solely for within-study variation. Therefore, the fixed-effect model was uniformly applied for direct pairwise comparisons. The incidence of NOD in the atorvastatin group (4.80%) was higher than that in the placebo group (4.74%), and the result was statistically significant [OR = 1.54, 95% CI (1.44, 1.66), p < 0.05] (Figure 4C). There were no significant statistical differences in the incidence of NOD between the inclisiran group and the placebo group [OR = 1.03, 95% CI (0.85, 1.25), p > 0.05] and between the evolocumab group and the placebo group [OR = 0.91, 95% CI (0.75, 1.10), p > 0.05] (Figures 4A,B).

### Reticular meta-analysis results

**FIGURE 5:**
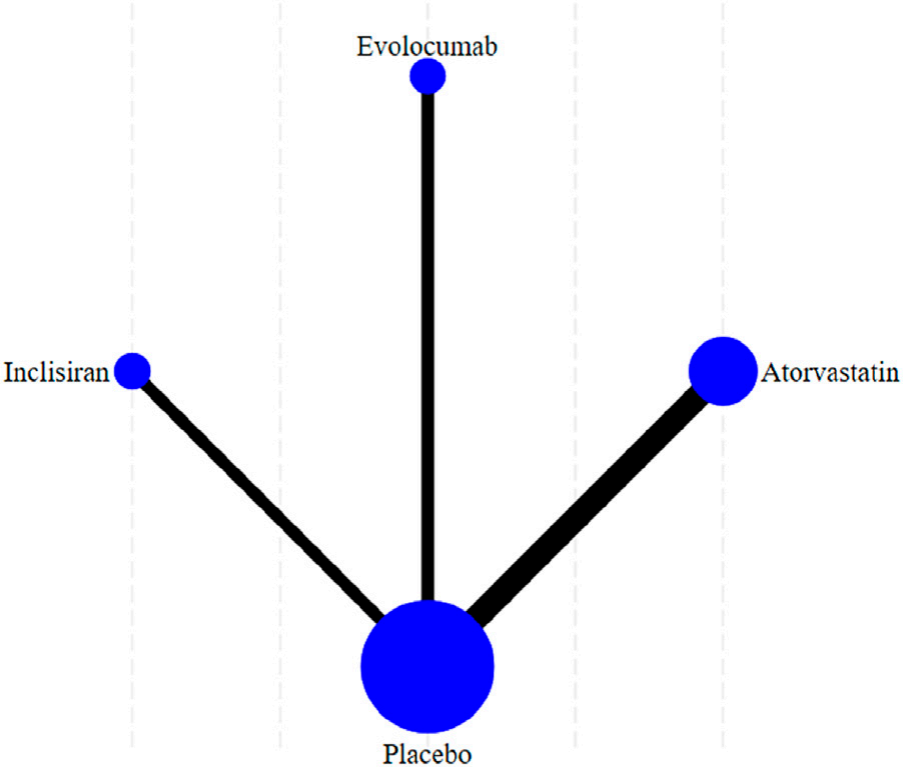
Network evidence plot of the incidence of NOD for each intervention.

Figure 5 shows the reticular diagram for each intervention. Each node (blue dot) in the figure represents an intervention; the size of each node represents the number of participants included in the intervention, and the larger nodes indicate a larger number of participants. The connection between the two points was a direct comparison between the two interventions, and the thicker the line segment, the more studies were compared between the two interventions. There were no connecting lines between different intervention methods, and indirect comparisons can be made using reticular meta-analysis.

**FIGURE 6:**
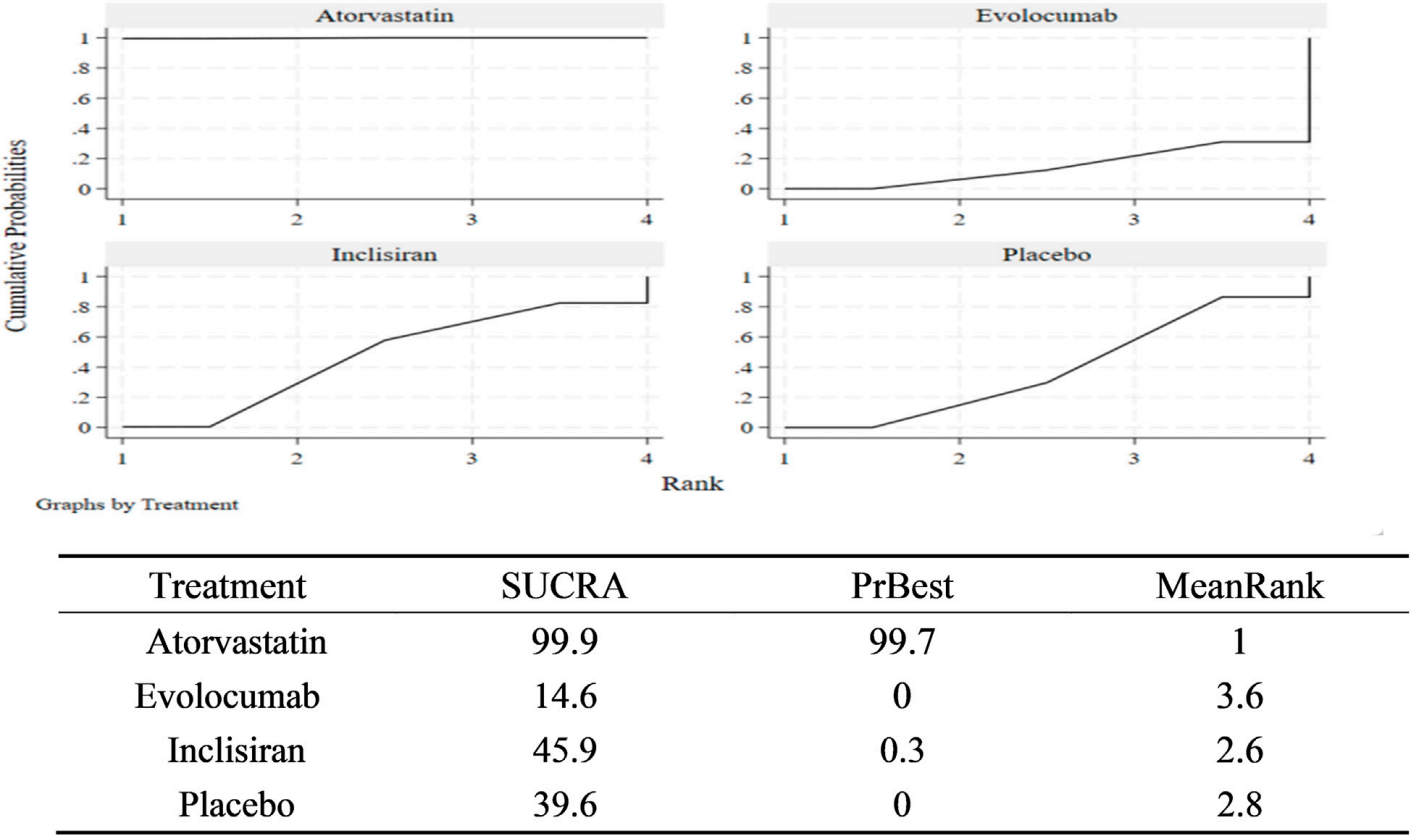
Area under the cumulative sequencing curve (SUCRA) of new diabetes incidence.

According to the match table (Table 5), the incidence of NOD was higher in the atorvastatin group than in the inclisiran group (OR = 1.42, 95% CI: 1.09–1.84), placebo group (OR = 1.46, 95% CI: 1.29–1.66), and evolocumab group (OR = 1.60, 95% CI: 1.23–2.08). However, the differences among the inclisiran, evolocumab, and placebo groups were not significant, indicating that inclisiran and evolocumab may not increase the incidence of NOD. According to the SUCRA chart (Figure 6), the incidence of NOD under the three interventions was ranked as follows: atorvastatin group (SUCRA = 99.9%)> inclisiran group (SUCRA = 45.9%)> placebo group (SUCRA = 39.6%)> evolocumab group (SUCRA = 14.6%).

**TABLE 5:** Table for the incidence of new-onset diabetes.

NOD			
Atorvastatin			
1.42 (1.09, 1.84)	Inclisiran		
1.46 (1.29, 1.66)	1.03 (0.82, 1.30)	Placebo	
1.60 (1.23, 2.08)	1.13 (0.82, 1.56)	1.09 (0.87, 1.38)	Evolocumab

**FIGURE 7:**
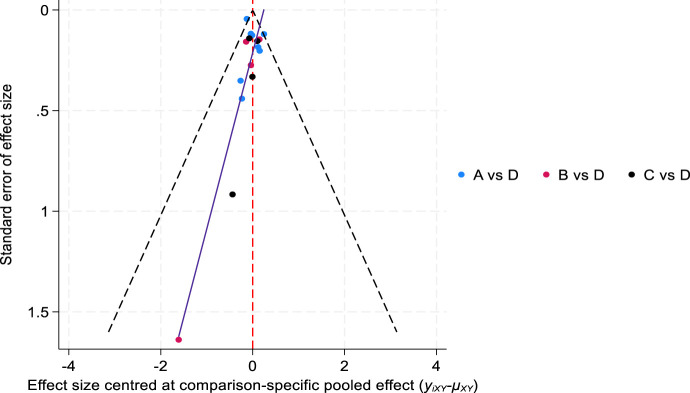
Comparison of the incidence of new-onset diabetes-corrected funnel plot. (**(A)** atorvastatin, **(B)** inclisiran, **(C)** evolocumab, and **(D)** placebo).

Comparison of the analysis results of new diabetes incidence data-corrected funnel plot showed that the included studies were centrally distributed, basically within the funnel, and symmetrically distributed, indicating that the included studies were less likely to have publication bias (Figure 7).

## Discussion

Inclisiran has been on the market for a relatively short time, so continuous monitoring of long-term AEs should be maintained during its clinical use. Whether there is a disease correlation between NOD and dyslipidemia remains a question. As future research delves deeper into the pathophysiology of these two diseases, new discoveries may emerge. Some relatively rare clinical and epidemiological AE data are better accessible and evaluated in the real world than in registry trials (Sisi et al., 2022). Global pharmacovigilance study plays an important role in tracking differences in the safety profiles of the same class of therapeutic agents (Goldman et al., 2022), but causal inference is somewhat challenging due to the nature of the adverse reactions. Meta-analysis of RCTs provides ways to address causality information in the FAERS report that has not been validated in science or otherwise. In this study, we combined the advantages of both approaches to provide a systematic, evidence-based approach to assess the association of inclisiran with NOD and hyperglycemia.

Our pharmacovigilance analysis showed that inclisiran had no significant impact on AEs of type 2 diabetes mellitus [ROR 0.95, 95% CI (0.31–2.96)] and impaired glucose tolerance [ROR 6.25, 95% CI (2.60–15.05)]. In contrast, atorvastatin was highly associated with AEs of type 2 diabetes mellitus [ROR 195.03, 95% CI (188.86–201.41)] and IGT [ROR 9.11, 95% CI (7.38–11.23)]. However, in the FAERS database, consumers and lawyers reported more adverse Ingram (57.04%) than health professionals (42.79%), which is potentially unbalanced, and bias and differences should be interpreted with caution. Based on the results of the pharmacovigilance analysis, we introduced a meta-analysis to further explore causality, as clinical trials have addressed these safety issues reported by health professionals. We extracted 16 studies from published articles to quantify the risk of adverse drug events. Our findings are consistent with the diabetes panel of the National Lipid Association Expert Panel on evidence that statins increase the risk of NOD (Maki et al., 2014). However, there was no statistically significant difference among the inclisiran, evolocumab, and placebo groups (as shown in Table 5).

Notably, patients treated with atorvastatin had a higher risk of NOD, but there were no significant changes in glycemic control and HbA1c in some patients (Angelidi et al., 2018), which was also validated in the FAERS database. Our retrospective query of the FAERS database found that atorvastatin treatment was not significantly associated with abnormal glycemia and elevated HbA1c. Statins moderately accelerate the time to diabetes diagnosis, and this risk is mainly limited to patients who are already at high risk of developing diabetes, such as those with impaired fasting glucose, metabolic syndrome, or raised HbA1c (Ridker et al., 2012).

Despite the clinical observation of a general association between LDL cholesterol and high risk of type 2 diabetes, it is far from certain that all interventions to lower LDL cholesterol increase the risk of type 2 diabetes, as the mechanisms of action of different lipid-regulating drugs may differ (Schmidt et al., 2017). Statins have been shown to increase the risk of diabetes, the underlying mechanism associated with reduced insulin secretion. The expression of LDLR in the pancreas, leading to increased LDL-C concentration in the pancreas, induces the lipotoxic effect of β cells and β-cell dysfunction, impairs insulin secretion, and promotes the development of type 2 diabetes mellitus (Laakso and Fernandes Silva, 2023; Takei et al., 2020). Regarding glucose metabolism and the risk of NOD, Langhi et al. studied the expression and function of PCSK 9 in islets and showed that PCSK 9 does not alter insulin secretion in mice (Langhi et al., 2009). However, some evidence from the Mendelian randomization study suggests that certain genetic PCSK 9 variants associated with low LDL-C levels are associated with a 29% increased risk of type 2 diabetes (Schmidt et al., 2017). Glodman et al. showed that PCSK 9 inhibitors are associated with increased reporting of hyperglycemic AEs, mainly with mild hyperglycemia (Goldman et al., 2022). The increased risk of NOD (hyperglycemia) was not observed in the longer trial of the ORION-1 study (Ray et al., 2023). Therefore, the existing studies are not sufficient to demonstrate the role of PCSK 9 and PCSK 9 monoclonal antibodies in the disruption of islet function. Moreover, the results of a *post hoc* pooled analysis of the phase-3 randomized trials ORION-9, ORION-10, and ORION-11 with a follow-up period of up to 540 days confirmed that inclisiran was well tolerated and did not increase the risk of NOD compared with the placebo group (Leiter et al., 2024).

In FAERS, we ranked the signal intensity of AEs in the top 30 PTs. The results indicate that evolocumab lacked administration/injection site rotation (ROR = 38.49/19.89; IC_025_ = 4.91/4.12) and injection fear (ROR = 14.33; IC_025_ = 3.70). Inclisiran reported no such obvious AEs compared with evolocumab (Supplementary Material S1). Therefore, when inclisiran is used twice a year and combined with lower rates of AEs, these give it higher treatment adherence than evolocumab (Scicchitano et al., 2021). However, given the short time of marketing of inclisiran, as with any new substance, the potential off-target effects and long-term safety of the drug should be closely monitored (Wolowiec et al., 2023). This needs to be confirmed by in-depth and large-scale long-term follow-up studies, especially with regular blood glucose monitoring in high-risk groups, and a comprehensive assessment of risk factors and possible drug interactions appears very important (Tosur et al., 2020).

## Limitations

Although pharmacovigilance and RCTs are complementary in an integrated study design, our study still has some limitations. First, the FAERS database is a global spontaneous reporting system, with nonmedical professionals expanding reporting coverage, but it has unstandardized data quality, possibly with reporting bias and substantial missing data. Second, the pharmacovigilance analysis failed to verify the causal relationship between inclisiran and AEs, as the disproportionate analysis provided only an estimate of signal strength and could neither quantify the risk nor determine causality (Chen et al., 2024). Finally, most RCT studies were not designed to study the relationship between inclisiran and NOD, which may lead to heterogeneity of results. Moreover, another important point is the potential impact of the COVID-19 pandemic (e.g., infection and healthcare access disruptions) on disease control and AE reporting during our study period (2021–2024). This is a recognized limitation of our study.

## Conclusion

Through a systematic evaluation of integrated pharmacovigilance and RCTs, our results showed that inclisiran may carry a lower risk of NOD than atorvastatin. However, inclisiran is a newly approved drug that has been available for a short duration in recent years, and larger long-term follow-up studies are needed to confirm these findings.

## Data Availability

The raw data supporting the conclusions of this article will be made available by the authors, without undue reservation.
