# Supplementary material for: Systemic evaluation of inclisiran on the risk of new-onset diabetes and hyperglycemia compared to evolocumab and atorvastatin
Source: Front Pharmacol. 2025 Jul 15;16:1554631. doi: 10.3389/fphar.2025.1554631 (PMC12303805; doi:10.3389/fphar.2025.1554631)
Supplement: Supplementary file 1 [file Table1.docx]

**Top 30 preferred language for inclisiran-positive signal intensity (PT)**

Table S1 Sequencing of signal intensity of adverse events to inclisiran (top 30 of PT)

| **Preferred language** | **Preferred Terms** | **Number of cases (Case)** | **ROR (95% CI)** | **IC(IC-2SD)** |
| --- | --- | --- | --- | --- |
| Lower in low-density lipoprotein | Low density lipoprotein decreased | 43 | 286.84(207.21-397.08) | 7.92(4.75) |
| Low-density lipoprotein was increased | Low density lipoprotein increased | 170 | 228.97(194.75-269.21) | 7.62(6.28) |
| Reduced blood cholesterol | Blood cholesterol decreased | 49 | 230.20(170.51-310.78) | 7.65(4.89) |
| Lipoprotein (a) was increased | Lipoprotein (a) increased | 8 | 277.39(130.89-587.87) | 7.88(2.08) |
| LDL | Low density lipoprotein | 3 | 341.60(98.15-1188.90) | 8.14(0.39) |
| The HDL was elevated | High density lipoprotein increased | 13 | 120.62(68.61-212.04) | 6.81(2.85) |
| Blood triglycerides were decreased | Blood triglycerides decreased | 6 | 151.87(65.72-350.97) | 7.12(1.60) |
| HDL | High density lipoprotein decreased | 18 | 78.10(48.64-125.40) | 6.22(3.26) |
| LDL abnormalities | Low density lipoprotein abnormal | 8 | 86.80(42.60-176.88) | 6.36(2.05) |
| Elevated blood triglycerides were observed | Blood triglycerides increased | 74 | 49.98(39.62-63.05) | 5.59(4.55) |
| gangrenous cellulitis | Cellulitis gangrenous | 3 | 95.65(29.83-306.72) | 6.50(0.46) |
| Non-HDL cholesterol elevation | Non-high-density lipoprotein cholesterol increased | 3 | 67.36(21.21-213.89) | 6.01(0.45) |
| Exception of lipids | Lipids abnormal | 5 | 49.52(20.33-120.62) | 5.59(1.24) |
| Abnormal blood cholesterol | Blood cholesterol abnormal | 19 | 27.56(17.50-43.39) | 4.76(2.90) |
| Blood triglycerides were abnormal | Blood triglycerides abnormal | 5 | 38.89(16.01-94.45) | 5.25(1.21) |
| Elevated blood cholesterol | Blood cholesterol increased | 90 | 16.11(13.07-19.84) | 3.98(3.46) |
| myalgia | Myalgia | 268 | 13.92(12.33-15.73) | 3.75(3.50) |
| Rashes at the injection site | Injection site rash | 56 | 14.67(11.27-19.10) | 3.85(3.16) |
| Elevated lipids | Lipids increased | 6 | 24.35(10.87-54.54) | 4.58(1.38) |
| unstable angina pectoris | Angina unstable | 9 | 20.60(10.67-39.76) | 4.35(1.87) |
| Injection site discomfort | Injection site discomfort | 24 | 15.24(10.19-22.80) | 3.91(2.69) |
| Injection site pain | Injection site pain | 356 | 9.20(8.27-10.23) | 3.14(2.96) |
| Injection site reaction | Injection site reaction | 70 | 10.20(8.06-12.91) | 3.33(2.81) |
| Vesicles at the injection site | Injection site vesicles | 10 | 12.04(6.46-22.44) | 3.58(1.71) |
| Body pain | Pain in extremity | 256 | 6.70(5.91-7.58) | 2.70(2.49) |
| arthralgia | Arthralgia | 407 | 6.17(5.58-6.81) | 2.57(2.40) |
| The injection site was discolored | Injection site discolouration | 13 | 9.57(5.55-16.52) | 3.25(1.79) |
| bronchitis | Bronchitis | 59 | 6.80(5.26-8.79) | 2.75(2.25) |
| carotid artery stenosis | Carotid artery stenosis | 4 | 13.26(4.95-35.48) | 3.72(0.64) |
| Musculoskeletal discomfort | Musculoskeletal discomfort | 20 | 7.58(4.88-11.76) | 2.91(1.89) |

**Top 30 Pwords for Evolocumab (PT)**

Table S2 Sequencing of signal intensity of adverse events to Evolocumab(top 30 of PT)

| **Preferred language** | **Preferred Terms** | **Number of cases (Case)** | **ROR(95% CI)** | **IC(IC-2SD)** |
| --- | --- | --- | --- | --- |
| Lower low-density lipoprotein | Low density lipoprotein decreased | 598 | 217.36(190.88-247.51) | 6.38(6.04) |
| The device is difficult to use | Device difficult to use | 20599 | 140.39(137.67-143.17) | 6.05(6.02) |
| Product discard in the wrong way | Incorrect disposal of product | 1134 | 129.78(119.60-140.84) | 6.05(5.86) |
| The device is omitted to be administered | Drug dose omission by device | 15737 | 83.72(82.05-85.42) | 5.64(5.61) |
| LDL abnormalities | Low density lipoprotein abnormal | 134 | 82.08(66.19-101.79) | 5.68(4.93) |
| Reduced blood cholesterol | Blood cholesterol decreased | 382 | 64.38(56.98-72.75) | 5.45(5.13) |
| Wrong use of the intercepted product | Intercepted product administration error | 1708 | 55.94(52.87-59.20) | 5.31(5.19) |
| Product communication issues | Product communication issue | 1196 | 53.44(49.97-57.15) | 5.26(5.12) |
| Apolipoprotein abnormalities | Lipoprotein abnormal | 4 | 177.96(39.83-795.18) | 6.26(0.59) |
| Accidental exposure to the product | Accidental exposure to product | 13259 | 35.11(34.43-35.80) | 4.75(4.72) |
| Low-density lipoprotein was increased | Low density lipoprotein increased | 812 | 34.87(32.28-37.67) | 4.80(4.64) |
| Lack of administration-site rotation | Lack of administration site rotation | 47 | 38.49(27.82-53.25) | 4.91(3.76) |
| Lipoprotein (a) was increased | Lipoprotein (a) increased | 25 | 39.73(25.42-62.09) | 4.95(3.21) |
| Lipoprotein (a) is abnormal | Lipoprotein (a) abnormal | 3 | 100.10(22.40-447.28) | 5.85(0.15) |
| Occupational exposure to the product | Occupational exposure to product | 788 | 23.38(21.68-25.23) | 4.32(4.18) |
| Device administration dose was incorrect | Incorrect dose administered by device | 2179 | 20.96(20.03-21.93) | 4.18(4.10) |
| revascularization | Revascularisation procedure | 6 | 44.49(17.66-112.08) | 5.07(1.33) |
| Product preparation error | Product preparation error | 1815 | 18.24(17.37-19.16) | 4.01(3.92) |
| Lack of injection-site rotation | Lack of injection site rotation | 147 | 19.89(16.72-23.65) | 4.12(3.72) |
| Arterial revascularization | Arterial revascularisation | 3 | 66.74(16.69-266.85) | 5.49(0.19) |
| High-density lipoprotein abnormalities | High density lipoprotein abnormal | 17 | 28.01(16.61-47.26) | 4.54(2.63) |
| Technical errors during product use | Wrong technique in product usage process | 17790 | 16.57(16.31-16.84) | 3.81(3.79) |
| Incorrect device use | Device use error | 2988 | 16.66(16.04-17.31) | 3.89(3.82) |
| The product gave an incorrect dose | Incorrect dose administered by product | 121 | 18.70(15.46-22.62) | 4.05(3.60) |
| Error in equipment distribution | Device dispensing error | 97 | 18.69(15.11-23.11) | 4.05(3.52) |
| Conditions that can lead to medication errors  Or information | Circumstance or information capable of leading to medication error | 2170 | 14.69(14.05-15.36) | 3.73(3.65) |
| Injection fear | Fear of injection | 460 | 14.33(13.02-15.78) | 3.70(3.52) |
| External medication site mass | Application site mass | 27 | 18.58(12.42-27.79) | 4.04(2.82) |
| Blood stasis at the injection site | Injection site bruising | 3851 | 11.72(11.34-12.11) | 3.42(3.37) |
| Separation of the blood components | Apheresis | 5 | 29.02(11.03-76.32) | 4.59(1.01) |

**Top 30 preferred words for atorvastatin positive signal intensity (PT)**

Table S3 Sequencing of signal intensity of adverse events to Atorvastatin (top 30 of PT)

| **Preferred language** | **Preferred Terms** | **Number of cases (Case)** | **ROR(95% CI)** | **IC(IC-2SD)** |
| --- | --- | --- | --- | --- |
| Progesterolone deficiency | Pregnenolone deficiency | 11 | 3346.25(432.00-25919.6) | 8.13(2.37) |
| Immune-mediated myositis | Immune-mediated myositis | 1059 | 355.92(325.71-388.93) | 7.36(7.04) |
| Necrotizing myositis | Necrotising myositis | 312 | 338.60(288.16-397.87) | 7.33(6.53) |
| Lecithin-cholesterol acyltransferase deficiency | Lecithin-cholesterol acyltransferase deficiency | 8 | 1216.79(258.38-5730.14) | 7.93(1.82) |
| Corticosterone was reduced | Blood corticosterone decreased | 6 | 1825.15(219.72-15160.8) | 8.03(1.28) |
| Autoimmune myositis | Autoimmune myositis | 112 | 277.23(214.60-358.13) | 7.18(5.66) |
| Type 2 diabetes | Type 2 diabetes mellitus | 6168 | 195.03(188.86-201.41) | 6.85(6.78) |
| Infant cervical fibromatosis | Fibromatosis colli of infancy | 7 | 425.87(135.16-1341.86) | 7.48(1.64) |
| Acanthocytic anemia | Spur cell anaemia | 21 | 212.96(121.93-371.96) | 6.97(3.50) |
| The amount of bile discharged | Bile output | 5 | 380.24(102.10-1416.03) | 7.41(1.05) |
| Edge erythema | Erythema marginatum | 6 | 304.19(98.10-943.20) | 7.25(1.39) |
| Congenital diaphragmatic anomaly | Congenital diaphragmatic anomaly | 6 | 260.74(87.62-775.86) | 7.14(1.41) |
| Glycogen cumulative disease type V | Glycogen storage disease type V | 9 | 195.56(84.64-451.81) | 6.90(2.13) |
| Anti-SRP antibody was positive | Anti-SRP antibody positive | 3 | 456.28(76.24-2730.76) | 7.52(0.11) |
| factitial hypoglycemia | Pseudohypoglycaemia | 6 | 165.92(61.36-448.66) | 6.75(1.44) |
| muscular death | Muscle necrosis | 81 | 77.53(60.74-98.96) | 5.95(4.80) |
| Traumatic coma | Traumatic coma | 7 | 141.96(57.88-348.18) | 6.60(1.71) |
| Myoglobinemia | Myoglobinaemia | 11 | 115.39(57.64-230.99) | 6.39(2.46) |
| Abnormal ambulatory blood pressure | Blood pressure ambulatory abnormal | 4 | 202.79(57.22-718.64) | 6.93(0.71) |
| Sword dented | Xyphoid retraction | 5 | 168.99(56.63-504.27) | 6.77(1.12) |
| Jessner Lymphocyte infiltration | Jessners lymphocytic infiltration | 5 | 168.99(56.63-504.27) | 6.77(1.12) |
| Nudites nipple stenosis | Papilla of Vater stenosis | 6 | 140.40(53.36-369.38) | 6.59(1.45) |
| multiple myositis | Polymyositis | 148 | 62.77(52.59-74.93) | 5.70(5.02) |
| The urinary albumin / creatinine ratio was decreased | Urine albumin/creatinine ratio decreased | 6 | 130.37(50.10-339.26) | 6.52(1.45) |
| myonosus | Myopathy | 644 | 53.15(48.87-57.80) | 5.50(5.28) |
| toxic myopathy | Myopathy toxic | 55 | 60.42(45.24-80.70) | 5.66(4.33) |
| Acquired oblique cephaly | Acquired plagiocephaly | 7 | 101.40(43.10-238.53) | 6.25(1.72) |
| Frailty of eye muscles | Ocular myasthenia | 31 | 62.88(42.71-92.57) | 5.71(3.77) |
| Cholestasis itching | Cholestatic pruritus | 5 | 117.00(41.71-328.18) | 6.41(1.15) |
| Abnormalities in hepatitis A antibodies | Hepatitis A antibody abnormal | 4 | 135.19(41.63-439.02) | 6.55(0.75) |
